# Utilisation of virtual non-contrast images and virtual mono-energetic images acquired from dual-layer spectral CT for renal cell carcinoma: image quality and radiation dose

**DOI:** 10.1186/s13244-021-01146-8

**Published:** 2022-01-24

**Authors:** Xiaoxiao Zhang, Gumuyang Zhang, Lili Xu, Xin Bai, Xiaomei Lu, Shenghui Yu, Hao Sun, Zhengyu Jin

**Affiliations:** 1 Department of Radiology, Peking Union Medical College Hospital, Peking Union Medical College, Chinese Academy of Medical Sciences, Shuaifuyuan No.1, Wangfujing Street, Dongcheng District, Beijing, 100730 China; 2 CT Clinical Science, Philips Healthcare, Beijing, 100600 China

**Keywords:** Dual-layer spectral CT, Renal cell carcinoma, Image quality, Radiation dose

## Abstract

**Background:**

Renal cell carcinoma (RCC) is the most common renal malignant tumour. We evaluated the potential value and dose reduction of virtual non-contrast (VNC) images and virtual monoenergetic images (VMIs) from dual-layer spectral CT (DL-CT) in the diagnosis of RCC.

**Results:**

Sixty-two patients with pathologically confirmed RCC who underwent contrast-enhanced DL-CT were retrospectively analysed. For the comparison between true non-contrast (TNC) and VNC images of the excretory phase, the attenuation, image noise, signal-to-noise ratio (SNR) and subjective image quality of tumours and different abdominal organs and tissues were evaluated. To compare corticomedullary phase images and low keV VMIs (40 to 100 keV) from the nephrographic phase, the attenuation, image noise, SNR and subjective lesion visibility of the tumours and renal arteries were evaluated. For the tumours, significant differences were not observed in attenuation, noise or SNR between TNC and VNC images (*p* > 0.05). For the abdominal organs and tissues, except for fat, the difference in attenuation was 100% within 15 HU and 96.78% within 10 HU. The subjective image quality of TNC and VNC images was equivalent (*p* > 0.05). The attenuation of lesions in 40 keV VMIs and renal arteries in 60 keV VMIs were similar to those in the corticomedullary images (*p* > 0.05). The subjective lesion visibility in low keV VMIs is slightly lower than that in the corticomedullary images (*p* < 0.05). Using VNC and VMIs instead of TNC and corticomedullary phase images could decrease the radiation dose by 50.5%.

**Conclusion:**

VNC images and VMIs acquired from DL-CT can maintain good image quality and decrease the radiation dose for diagnosis of RCC.

## Key points

- VNC from a DL-CT system was used to replace TNC images in RCC.
- Low-keV nephrographic VMIs were used to provide enhancement information about RCC lesions.
- Two-phase scanning in RCC examinations can be used to reduce radiation dose.

## Background

Most of the malignant tumours found in kidney CT examination are renal cell carcinomas (RCCs), which account for nearly 85% of all renal cancers in adults [1–3]. The most common histology of RCC is clear cell renal cell carcinoma (ccRCC), which accounts for 70% to 80% [4]. CT scans have the advantages of short imaging time, few contraindications and high accuracy and are currently the most commonly used examination method for preoperative diagnosis and staging of RCC [5, 6]. For the best diagnosis and most accurate staging, the reference standard for dedicated multiphase renal cell carcinoma imaging consists of unenhanced phase, corticomedullary phase, nephrographic phase and excretory phase scans [7]. Multiphase scans can provide sufficient diagnostic information, including the baseline CT density value of the tumours, the degree and characteristics of lesion enhancement, the relationship between tumours and arteries as well as the extent of renal pelvis involvement [6]. However, in different scanning conditions, radiation dose of multiphase CT was found to be about 20–30 mSv, and studies have shown that the dose is associated with the risk of malignancy and loss of life expectancy [8–10]. For the patients with RCC, the 5-year survival among patients discovered without symptoms is up to 85% and among patients without metastases is now over 50% [1]. Hence radiation dose remains a concern for patients with RCC.

The application of dual-energy CT (DECT) technology makes it possible to reduce the scanning phases and at the same time meet the requirements of clinical diagnosis. The major dual-energy systems include dual-source dual-energy systems (dsDECT), single-source rapid KV switching systems (rsDECT) and dual-layer spectral CT (DL-CT) [11]. Compared to dsDECT and rsDECT, spectral separation of DL-CT, which uses a single high tube potential beam and layered or “sandwich” scintillation detectors, occurs at the detector level, after the X-ray beam has interacted with the patient [12]. The top layer selectively absorbs low-energy photons, while the high-energy photons penetrate this layer to the bottom layer. Hence, there is no need to set dual-energy scanning program in advance and potential inaccuracies related to spatial misregistration could be eliminated [11, 13]. DECT allows for the reconstruction of virtual non-contrast (VNC) and virtual monoenergetic images (VMIs) from a contrast-enhanced examination [14]. VNC images can provide baseline CT values just like true non-contrast (TNC) images. VMIs reconstructed at lower energies (40–70 keV) can be used to enhance the attenuation of iodine due to the increased photoelectric attenuation at energies approaching the K-edge of iodine (33.2 keV) [15].Therefore, it is reasonable to assume that VNC may represent an alternative to TNC and that low-keV VMIs generated from the nephrographic phase could compensate for the reduction in iodine concentration and provide roughly the same clinical diagnostic information as the corticomedullary phase images.

Although numerous phantom and human studies have advocated for the routine adoption of VNC in place of TNC images, a literature review indicated that relevant studies have not explored the application of VNC and VMI of DL-CT in diagnosing renal solid tumours, especially RCC. Hence, the aim of this retrospective study was to evaluate whether TNC images and corticomedullary phase images could be replaced by VNC images from excretory phase data and low keV VMIs from nephrographic phase data to reduce the radiation dose.

## Materials and methods

### Study population

Between October 2017 and November 2020, a total of 77 patients were histologically confirmed to have RCC after surgical treatment and had undergone a preoperative contrast-enhanced DL-CT in our hospital. The exclusion criteria were the absence of VNC images from the excretory phase or the absence of VMIs in the nephrographic phase and therapeutic interventions before the examination. Eight patients, two patients and five patients were excluded from the study due to lack of VNC images, VMI images and presence of prior therapeutic intervention, respectively.

The institutional review board approved the retrospective study, and informed patient consent was waived.

### CT protocol and image reconstruction

**Fig. 1:**
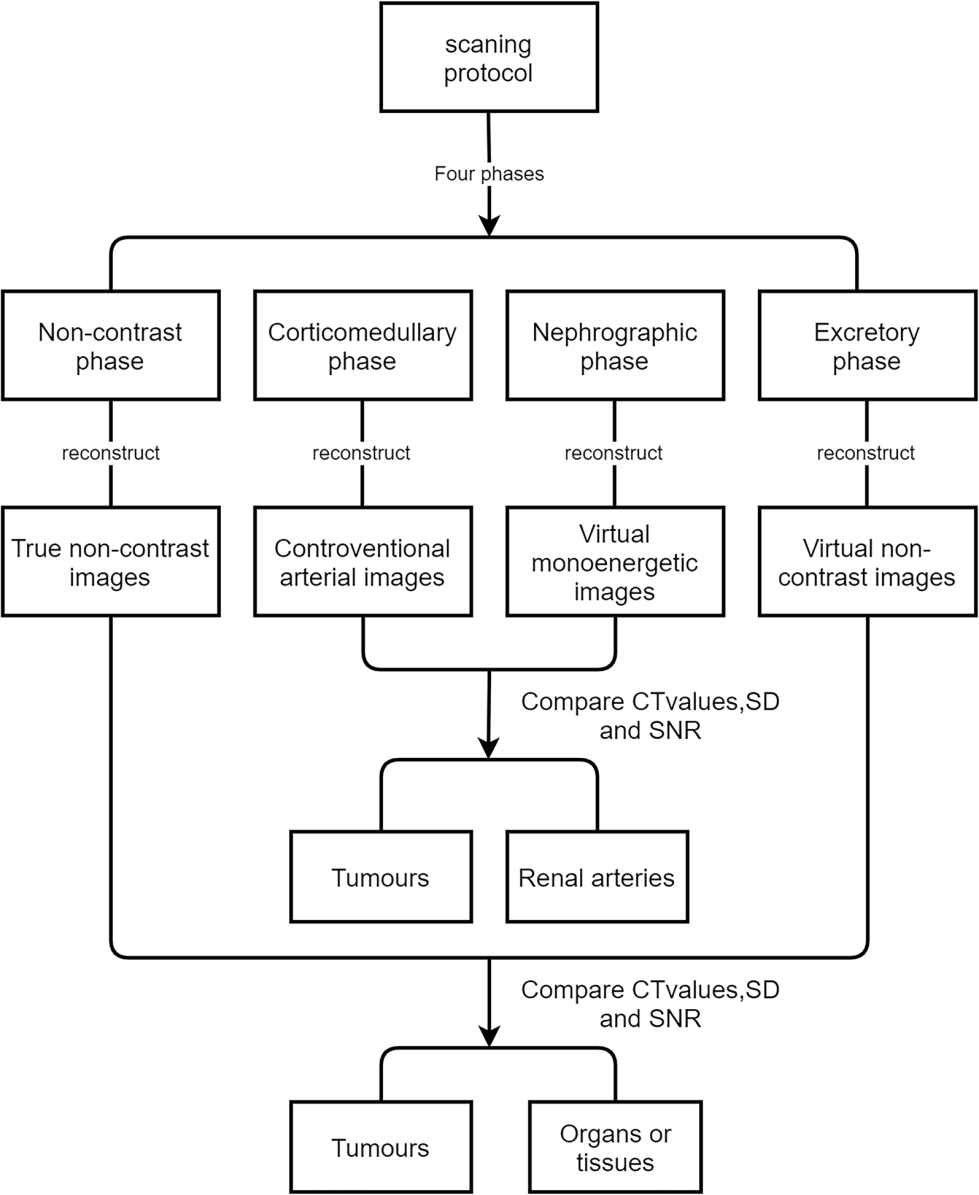
Flowchart of study design

All examinations were performed on a DL-CT (IQon spectral CT, Philips Healthcare) system. The scan ranged from the top of the liver to the bifurcation of the bilateral common iliac arteries. Four-phase images, including true non-enhanced phase, corticomedullary phase, nephrographic phase and excretory phase, were acquired. All examinations were performed at a tube potential of 120 kV and detector configuration of 64.0 × 0.625 mm with automatic tube current modulation. When performing enhanced examination, the abdominal aorta was detected by intelligent tracking. When the density of the aorta reached the threshold of 120 HU, the scan was triggered automatically. The nephrographic phase scan was started approximately 70 s later, and the excretory phase scan was started approximately 420 s later. Spectral database images (SBIs) were transferred to a workstation (IntelliSpace ®Portal Workstation, Philips Healthcare). The acquisition slice thickness of the four-phase images was 1 mm. Then, the VNC images from the excretory phase and TNC images were reconstructed with a 5-mm slice thickness, and VMIs with a 1-mm slice thickness were generated between 40 and 100 keV by a linear combination of photoelectric and Compton images (Fig. 1). The images were transmitted to the picture archiving and communication system (PACS, Centricity, GE Medical Systems, Milwaukee, Wisconsin, USA) for further evaluation.

### Quantitative analysis

For the TNC images and VNC images from the excretory phase, we compared the attenuation (CT value), noise (standard deviation, SD) and signal-to-noise ratio (SNR = mean attenuation/standard deviation) of the tumours as well as different organs and tissues. Those were measured by placing regions of interest (ROIs) on the target area in both TNC and VNC images. For the RCC lesions, 2 or 3 ROIs whose area is about 80–100 mm^2^ were placed at the level of the largest solid component of the tumours and the average value was taken. If calcification was observed in the RCC lesion, the number of calcifications in both TNC and VNC images was counted. The ROI was placed with avoiding calcified and necrotic components. The maximum length and short-axis diameter, which were measured on the same slice and perpendicular to the measurement of the "maximum length" of the tumour, were measured on the axial slice with the largest solid component of the tumours in the TNC, VNC, corticomedullary phase images and VMIs from nephrographic phase. For the organs and tissues, ROIs were delineated in the liver, spleen, aorta, bilateral kidneys, subcutaneous fat of the anterior abdominal wall, and right psoas muscle. The ROIs of the liver, spleen and aorta were placed at the level that the portal vein gives off the left and right hepatic branches, the ROIs of the bilateral kidneys were placed at renal hilum level and the ROIs of the right psoas muscle and subcutaneous fat of anterior abdominal wall were placed at the level immediately distal of the aortic bifurcation. Four ROIs were placed in the liver and two ROIs were placed in bilateral kidneys, respectively, and the mean values were taken. One ROI each was placed in spleen, aorta, right psoas muscle and subcutaneous fat, respectively. These measurements were repeated three times, and the average value was determined. The mean standard deviation of the measured values is the noise of each tissue and organ. A tolerance interval of ± 15 HU showed that TNC and VNC images were interchangeable techniques, while a more restrictive tolerance interval of ± 10 HU was used for the analysis of the muscle [16]. Minimum interval of ± 5 HU showed the excellent consistency. Hence, the proportion of TNC and VNC difference in tolerance range was calculated.

For the VMIs, we compared the attenuation, noise and SNR (SNR = mean attenuation/standard deviation) of the tumours and bilateral renal arteries in the corticomedullary phase images and nephrographic phase VMIs from 40 to 100 keV by placing ROIs on the target area. For the RCC lesions, 2 or 3 ROIs were placed at the level of the largest solid component of the tumours, and the average value was determined as for the TNC and VNC images. The ROIs of the bilateral renal arteries were drawn on the slice that showed the renal arteries originating from the aorta, with an area of 15–25 mm^2^. The ROI was placed with avoiding calcified and necrotic components. Noise was obtained by averaging the standard deviation of the measured values. All the ROIs were drawn by the same radiologist to ensure that the ROI size and location were as similar as possible between the TNC and VNC images as well as between the corticomedullary phase images and low keV nephrographic phase VMIs.

### Qualitative analysis

Two independent radiologists with 3 and 14 years of experience subjectively assessed the image quality of each TNC and VNC dataset using a 5-point Likert scale, and they provided their own ratings. In addition, through objective quality assessments, if the CT value of the RCC lesions in VMIs of a certain level did not significantly differ from that in the corticomedullary phase images, then the two radiologists would subjectively evaluate the lesion visibility in these two sets of images using 5-point Likert scales and provide their own scores instead of reaching a consensus. The 5-point Likert scales were evaluated as follows: 5 = excellent, 4 = good, 3 = fair, 2 = poor, and 1 = unreadable [17]

### Radiation exposure

To assess radiation dose, the dose–length product (DLP) displayed on the scanner was recorded and the effective radiation dose (ED) was calculated. The ED is equivalent to DLP multiplied by the abdominal conversion coefficient (K = 0.015 mSv/mGy.cm). To assess the level of dose reduction in this study, the EDs of true non-enhanced phase and corticomedullary phase were compared with those of the nephrographic phase and excretory phase.

### Statistical analysis

Statistical analysis was performed using IBM SPSS Statistics 25 for Macintosh (SPSS, Inc., Chicago, IL, USA). The data were expressed as the mean ± SD. To evaluate the objective image quality, the CT attenuation, noise and SNR were compared between the TNC and VNC images with the Mann–Whitney U test. For the VMIs, the CT attenuation, noise and SNR of the kidney tumours and bilateral renal arteries were compared between the corticomedullary phase images and nephrographic VMIs from 40 to 100 keV (low keV VMIs) with the Mann–Whitney U test. Then, inter-technique agreement between the TNC and VNC images in the attenuation measurements was evaluated using the Bland–Altman diagram**.** For subjective image quality, the Wilcoxon signed-rank test was used to compare the quality. Then, interobserver agreement was evaluated using Cohen’s kappa values, which were interpreted as slight (< 0.20), fair (0.21–0.40), moderate (0.41–0.60), substantial (0.61–0.80) and almost perfect (0.81–1.00). Radiation doses were compared between the conventional modes by the Kolmogorov–Smirnov test.

A *p* value < 0.05 was regarded as statistically significant for all tests cited above.

## Results

Sixty-two consecutive patients with a mean age of 55.4 ± 11.5 years (range 27–77 years) fulfilled our inclusion criteria, and they included 45 men (55.0 ± 11.6 years; range 27–77 years) and 17 women (57.4 ± 11.4 years; range 40–76 years). The post-operative histopathological examination confirmed ccRCC in 51 cases, papillary renal cell carcinoma in 5 cases, chromophobe renal cell carcinoma in 3 cases and other types of RCC in 3 cases.

The maximum length and the short-axis diameter on TNC, VNC, corticomedullary phase images and VMIs from nephrographic phase were (47 ± 26 mm, 40 ± 21 mm), (46 ± 25 mm, 40 ± 21 mm), (47 ± 26 mm, 40 ± 21 mm) and (47 ± 26 mm, 40 ± 21 mm), respectively. Statistically significant differences in tumour size measurements were not observed among the four-phase images.

### Quantitative analysis of the TNC and VNC images from excretory phase data

The quantitative quality comparisons between the TNC and VNC images are shown in Table 1. There was no difference between VNC and TNC images in mean attenuation for the RCC lesions, liver, spleen, aorta (*p* > 0.05), whereas differences were statistically significant for the kidneys, subcutaneous fat and psoas muscle (*p* < 0.05). Except for fat, the mean difference between the TNC and VNC images was less than 3 HU for all tissue types.

**Table 1:** CT values, noise and SNR of different organs and tissues in the TNC and VNC images

	TNC	VNC	*p* value
*CT value (HU)*			
Tumour	33.66 ± 6.69	32.68 ± 9.29	0.357
Liver	55.34 ± 6.29	56.89 ± 5.29	0.172
Spleen	47.36 ± 3.30	48.35 ± 4.00	0.165
Kidney	31.16 ± 2.94	28.48 ± 3.95	< 0.05
Aorta	40.49 ± 4.59	40.24 ± 5.49	0.778
Muscle	49.23 ± 4.53	47.13 ± 3.86	0.004
Fat	− 110.24 ± 12.93	− 99.63 ± 7.12	< 0.05
*Noise (HU)*			
Tumour	9.64 ± 1.61	9.62 ± 2.64	0.449
Liver	8.33 ± 1.22	8.14 ± 1.68	0.288
Spleen	8.74 ± 1.43	8.09 ± 1.76	0.018
Kidney	9.13 ± 1.60	8.80 ± 1.87	0.313
Aorta	10.25 ± 1.74	10.26 ± 1.92	0.974
Muscle	10.97 ± 1.96	10.11 ± 1.92	0.040
Fat	7.07 ± 1.62	6.96 ± 1.93	0.043
*SNR*			
Tumour	3.56 ± 1.03	3.67 ± 1.61	0.579
Liver	6.89 ± 1.34	7.41 ± 1.37	0.079
Spleen	5.54 ± 1.01	6.35 ± 1.67	0.009
Kidney	3.51 ± 0.66	3.38 ± 0.95	0.091
Aorta	4.07 ± 0.73	4.07 ± 1.18	0.656
Muscle	4.64 ± 0.96	4.84 ± 1.07	0.389
Fat	− 16.54 ± 3.73	− 15.50 ± 4.21	0.113

There were no statistically differences in noise for lesions, liver, aorta and kidney (*p* > 0.05), whereas slightly lower noise values on VNC images compared to TNC images reached statistical significance for spleen, muscle and fat (*p* < 0.05; Table 1). Except for the spleen, the SNRs of organs and tissues were not significantly different on TNC and VNC images (*p* > 0.05). The slightly higher SNR of the spleen on the VNC images was statistically significant (*p* < 0.05).

**Fig. 2:**
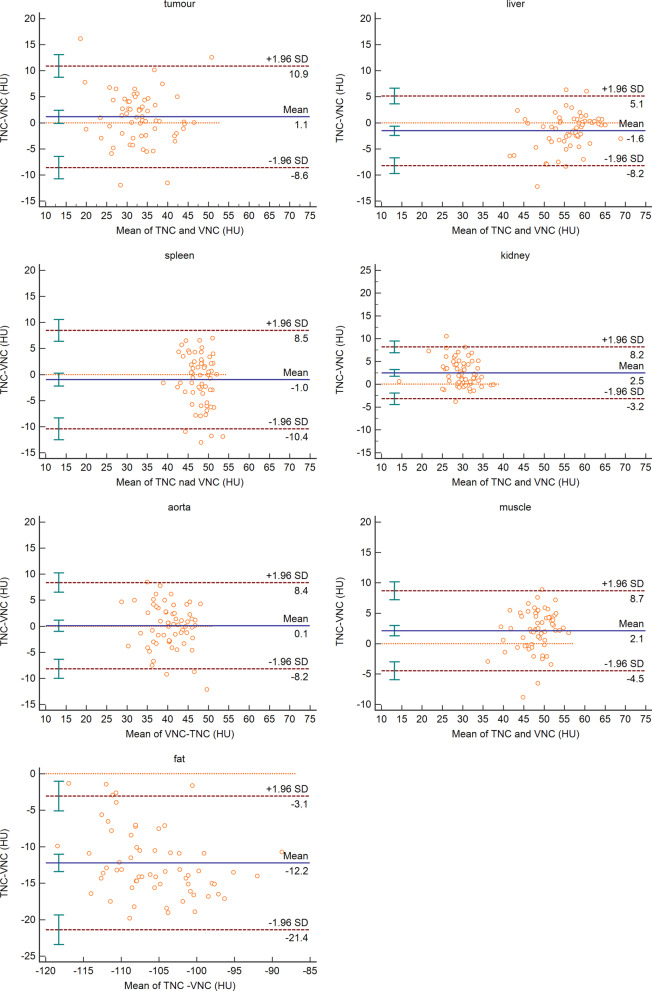
Bland–Altman diagrams. This figure shows the agreement in attenuation measurements between the true non-contrast (TNC) and virtual non-contrast (VNC) images for various organs and areas. Each orange circle represents the measurement results of the TNC and VNC images of each organ in each patient, the abscissa is the mean value of the TNC and VNC of this organ in this patient, and the ordinate is the difference of this result. The blue horizontal solid line in the middle represents the average of the differences. The two red horizontal dashed lines above and below the figure indicate the upper and lower limits of the 95% consistency limit

The percentage of difference measurements for each tissue lying within the tolerance intervals (5, 10 and 15 HU) is reported in Table 2. Except for fat, the measurement difference in CT values of all other tissues and organs between the two sets of images was 100% within 15 HU and 96.78% within 10 HU. The Bland–Altman diagrams for each tissue in terms of mean attenuation are reported in Fig. 2. In total, there were 94.56% of the measurements in the consistency boundary of tumours and each tissue.

**Table 2:** Differences in attenuation values between the TNC and VNC images

	[− 5 HU; + 5 HU] (%)	[− 10 HU; + 10 HU] (%)	[− 15 HU; + 15 HU] (%)
Tumour	80.65	91.93	100
Liver	82.26	98.39	100
Spleen	69.35	93.55	100
Kidney	79.03	98.39	100
Aorta	83.87	98.39	100
Muscle	74.19	100	100
Fat	11.29	24.19	70.97
All	68.66	86.57	95.85

**Fig. 3:**
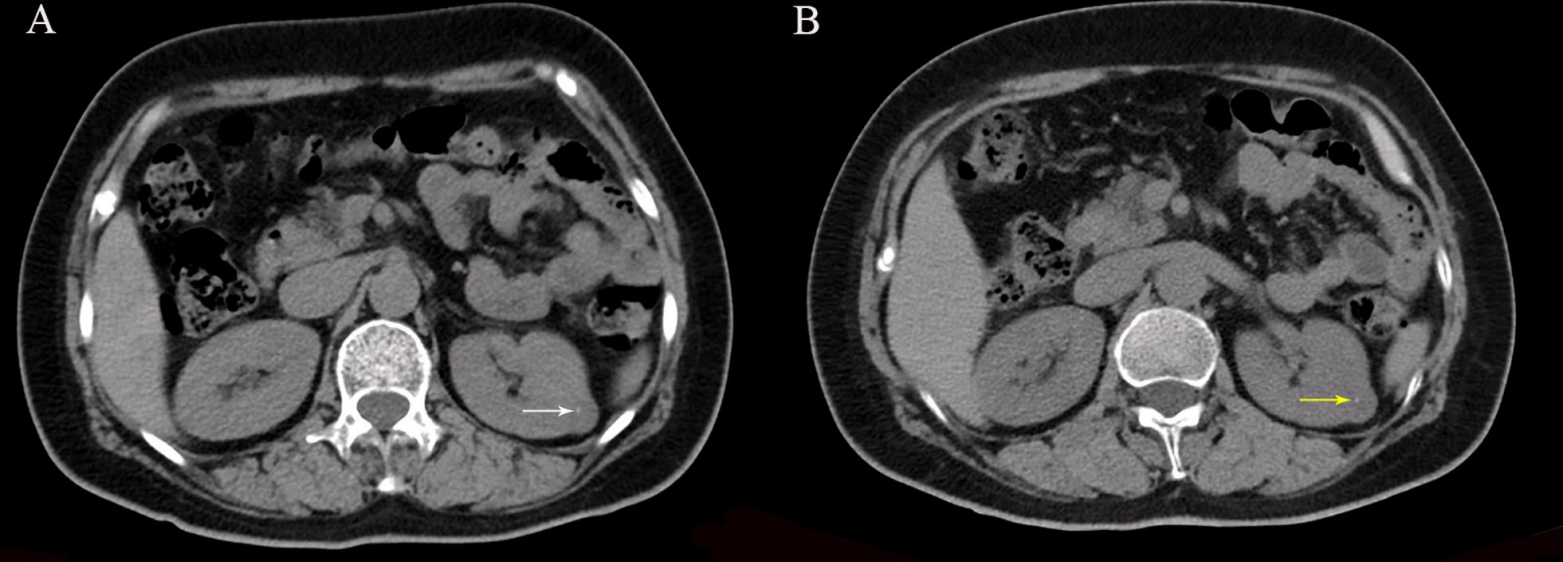
The images of a 56-year-old woman with clear cell carcinoma of the left kidney showed clear microscopic calcification on both TNC (**A**) and VNC images (**B**)

Calcification was found in five patients in the present study. A total of 17 calcifications with a minimum size of 2.5 mm were detected using the TNC images. All the calcification could be clearly observed in VNC images (Fig. 3).

### Quantitative analysis of corticomedullary phase images and VMIs from nephrographic phase data

**Fig. 4:**
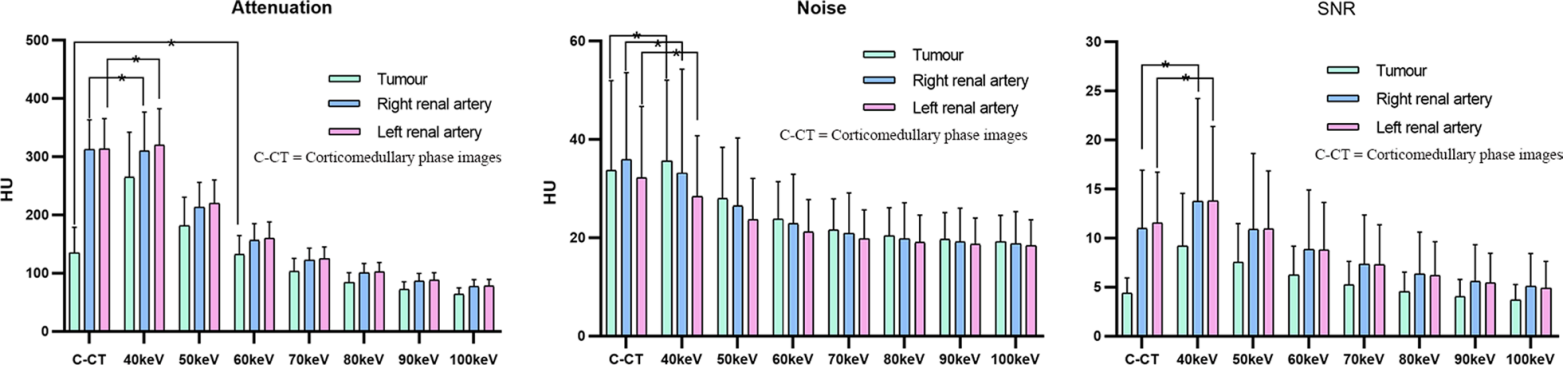
Mean attenuation, image noise and SNR (signal-to-noise ratio) of kidney tumours and renal arteries on corticomedullary phase images and nephrographic phase VMIs (virtual mono-energetic images) from 40 to 100 keV. From 40 to 100 keV, the mean attenuation, image noise and SNR decreased gradually. C-CT means corticomedullary phase images. *p > 0.05

Values of mean attenuation and noise and calculated SNR for RCC lesions and bilateral renal arteries in corticomedullary phase images and low keV VMIs (40–100 keV) reconstructed from nephrographic phase data are shown in Fig. 4. Values for tumour tissue obtained from 40 and 50 keV VMIs were higher and those from 70 to 100 keV were lower than that on corticomedullary phase images (*p* < 0.05), respectively. The values for both renal arteries obtained at 50–100 keV were significantly lower than those on the corticomedullary phase images (*p* < 0.05). Considering the VMIs of tumour tissue at 60 keV, the noise was significantly lower compared to that on the corticomedullary phase images (*p* < 0.05) and the SNR was significantly higher (*p* < 0.05). The noise and SNR of the bilateral renal arteries on the 40 keV VMIs were not significantly different from those on the corticomedullary phase images (*p* > 0.05).

### Qualitative analysis

**Fig. 5:**
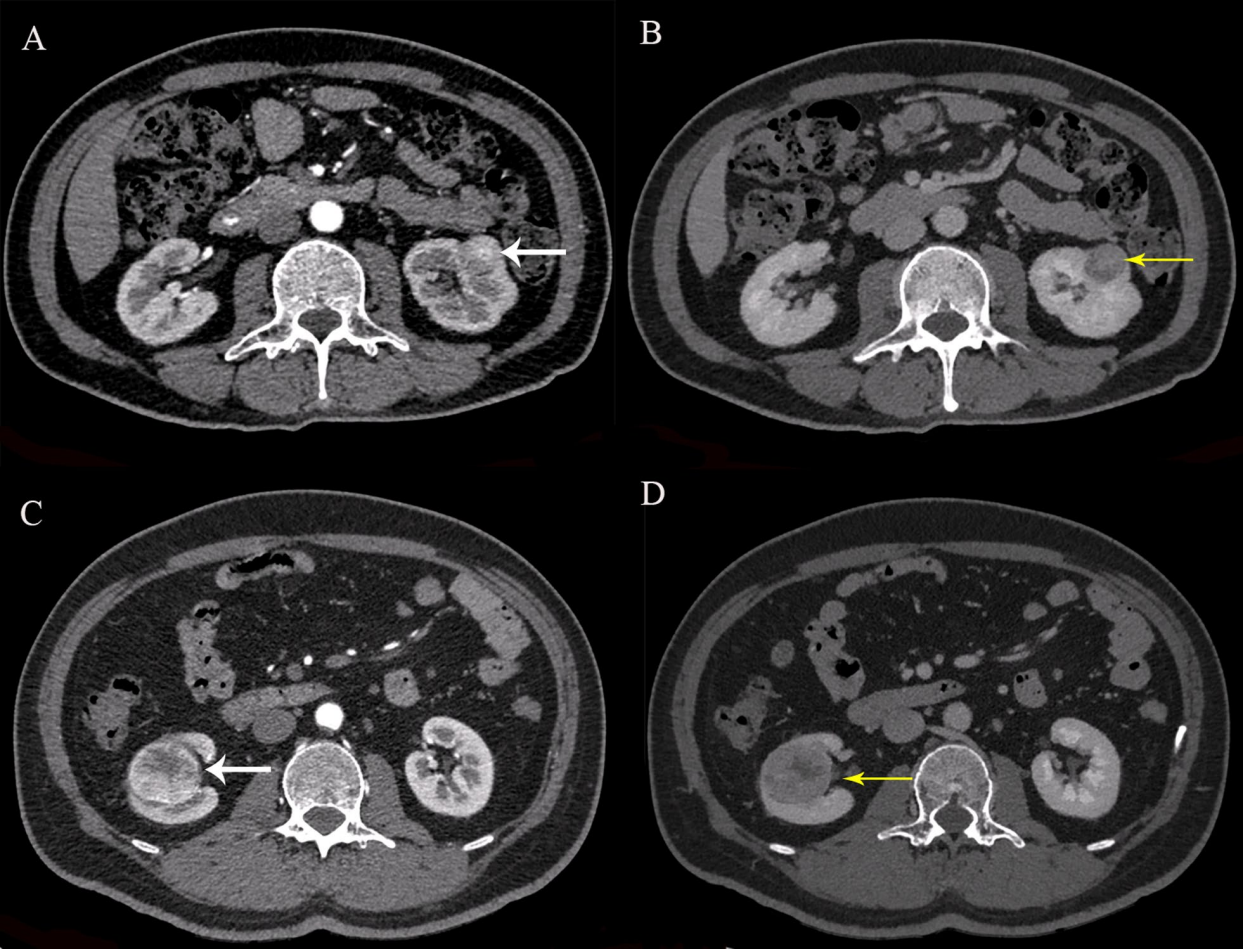
Images showed that a 58-year-old man had clear cell carcinoma of the left kidney with a diameter of 23.9 mm (**A**, **B**). The CT value of the lesion in the corticomedullary phase image (**A**) was similar to that of normal renal cortical enhancement. The lesion was not significant in the corticomedullary phase but could be clearly displayed on 60-keV nephrographic phase VMIs (virtual mono-energetic images) (**B**). Images showed that a 47-year-old man had clear cell carcinoma of the right kidney with a size of 41.4 mm × 35.0 mm (**C**, **D**). The lesion visibility was superior on corticomedullary phase images (**C**) than on 60 keV VMIs (**D**)

The qualitative results for the image quality and lesion visibility are shown in Table 3. For the VNC and TNC images, the quality scores were not significantly different (*p* > 0.05*)*. Between the two readers, there was perfect interobserver agreement for TNC images (Cohen’s kappa values = 0.828) and VNC images (Cohen’s kappa values = 0.850). Lesion visibility was subjectively evaluated between corticomedullary phase images and 60 keV VMIs from the nephrographic phase. The lesion visibility scores for the 60 keV VMIs were lower than those for the corticomedullary phase images (*p* < 0.05). Between the two readers, perfect interobserver agreement was observed for the corticomedullary phase images (Cohen’s kappa values = 0.891) and 60 keV VMIs (Cohen’s kappa values = 0.870). Readers 1 and 2 scored lesion visibility higher on VMI images in 10 and 9 of the 62 cases, respectively (Fig. 5). Readers 1 and 2 were consistent in assigning a score of only 3 for the VMI images in the same 5 cases. In addition, Readers 1 and 2 scored lesion visibility superior on corticomedullary phase images in 23 and 24 of the 62 cases (Fig. 5).

**Table 3:** Qualitative analysis of the image quality and lesion visibility

Group	Reader 1						Reader 2						Kappa value
Score*	1	2	3	4	5	Mean	1	2	3	4	5	Mean	
*Image quality*													
TNC images	0	0	0	10	52	4.84	0	0	0	11	51	4.82	0.828
VNC images	0	0	0	13	49	4.79	0	0	0	12	50	4.80	0.850
*Lesion visibility*													
Corticomedullary phase images	0	0	0	20	42	4.68	0	0	0	21	41	4.66	0.891
60kevVMI images	0	0	5	33	24	4.31	0	0	5	34	23	4.29	0.870

*The scores were obtained according to the Likert scale from 1 = unreadable to 5 = excellent

### Radiation dose

The ED for conventional scan model that included all four phases was 20.29 ± 7.36 mSv. The ED for the VNC + VMI scan model that only includes nephrographic and excretory phase was 10.08 ± 3.55 mSv, which was 50.32% lower than that of the conventional scan mode (*p* < 0.05).

## Discussion

In this study, a high agreement was observed between VNC and TNC images for histologically confirmed RCC lesions and other abdominal organs and tissues in DL-CT. A novel finding of this study is that VMIs from the nephrographic phase could provide almost as much information for clinical diagnosis (location, size, enhancement, relationship with renal arteries and lesion visibility) as corticomedullary phase images.

To explore the application of VNC images in RCC, the CT value, noise and SNR of tumours in TNC and VNC images were evaluated. Our study demonstrated that in the DL-CT system, the CT value, noise and SNR of the RCC lesions were not significantly different from those of the TNC images. In previous studies, Merer et al. judged that in the dsDECT and rsDECT systems, kidney lesions were in good agreement between VNC and TNC images. However, most of the kidney lesions in that study were cystic lesions, with a relatively small proportion of renal parenchymal tumours, and most of the lesions were not confirmed by pathological results [18]. Ma et al. explored the consistency of the CT values of RCC lesions in VNC and TNC images but did not extend the study to DL-CT imaging systems [19]. DL-CT has unique advantages, the most prominent of which is that the energy scan does not need to be set in advance and spatial registration errors do not occur [20]. In our study, there were 17 microcalcifications of 5 lesions that could be observed well on VNC. Hence, our study demonstrates the applicability of VNC instead of TNC in examining RCC lesions using DL-CT systems.

To determine whether VNC can replace TNC in the examination of patients with RCC, in addition to evaluating of renal lesions, other abdominal organs should also be evaluated. In our study, the CT values of abdominal tissues and organs were not significantly different except kidney, muscle and fat. The average difference between the organ and tissue measurements did not exceed 3 Hu with the exception of fat. The noise of the VNC images was equal to or lower than that of the TNC images, and the SNR was equal to or higher than that of TNC images. In the prior study, a tolerance interval of ± 15 HU indicated that the TNC images and VNC images were interchangeable [16, 21]. In our study, except for fat, the measurement differences in CT values of all tissues and organs were within 15 HU. In the prior study, Jamali et al. [16] showed that the difference between TNC and VNC attenuation values was less than 15 HU in 98.3%, while Ananthakrishnan et al. showed that it was less than 15 HU in 93.3% of all measurements [17]. Our results are better than the finding of these two studies except for fat, which may be due to different CT scanning schemes and image reconstruction techniques, which need additional studies to verify. The noise in the VNC images for each organ was similar to that of the TNC images, and the subjective evaluation indicated that the VNC images had similar quality scores as the TNC images. These results are also similar to those of previous studies [16, 17, 21]. The findings suggest that the overall quality of the VNC images is not lower than that of the TNC images, which provides another basis for replacing TNC with VNC.

In the phase selection process, the VNC images in our study are generated from an excretory phase other than nephrographic phase, which differ from that in previous studies [16, 21]. A previous study showed that the excretory phase may have high-density artefacts in the renal collecting system because of the excessive concentration of contrast agent in the renal collecting system [19]. In this study, the lower CT value of the kidney in VNC images than in TNC images may be related to the above phenomenon. However, the previous study showed that the CT value in VNC generated by nephrographic images also decreased [16, 22]. The results of our study showed that VNC images from the excretory phase can also generate images of similar quality to TNC images, suggesting that VNC phase selection could be more flexible in practical clinical applications.

This study also evaluated whether low keV VMIs from the nephrographic phase could provide sufficient clinical diagnostic information similar to corticomedullary images in the observation of RCC. VMIs have generally been used to reduce the amount of contrast material or to salvage CT examinations with poor contrast because the image contrast can be increased with the same iodine dose or the same contrast can be achieved with a reduced iodine load with low-keV VMIs [23–25]. Zopfs et al. showed that low-keV virtual monoenergetic imaging reconstructions of the excretory phase of urothelial carcinoma could replace venous phase images to reduce the radiation dose [26]. However, few feasibility studies have evaluated whether VMIs from the nephrographic phase can replace corticomedullary images. In our study, the mean attenuation of tumours in 60 keV VMIs and bilateral renal arteries in 40 keV VMIs from the nephrographic phase were similar to that in corticomedullary phase images, and all RCC lesions could be observed in the VMIs. Our study provides new insights for the clinical application of VMI images. Low-keV VMIs from the nephrographic phase could provide tumour enhancement information and arterial conditions such as the corticomedullary phase. Although the subjective score of lesion visibility on the low keV VMIs of nephrographic phase was slightly lower than that on corticomedullary phase images, the average score was more than 4, which does not affect clinical diagnosis. We may draw the conclusion that low-keV VMI techniques can be used to infer enhanced CT values of the tumour in the corticomedullary phase, even if we only obtain nephrographic images. We found that when using VNC instead of TNC and further using the VMIs instead of the corticomedullary phase images, the radiation dose reduction could reach 50%.

Our study also has several limitations. First, the sample size was small and all patients were from one institution. Thus, more cases are required and multicentre studies should be performed. Second, VNC reconstructions were only obtained from the excretory phase data. Although our results showed that the image quality generated in this phase was not different from that of the TNC, the exploration of DL-CT VNC images will be more comprehensive if enhancement studies of the other two phases are added. Third, all the tumours had RCC lesions, and a detection rate or sensitivity-specific evaluation of RCC lesions were not performed when using VNC images and VMIs; therefore, further studies will be conducted in future.

## Conclusion

In conclusion, our study demonstrated good agreement in the image quality for the evaluation of tumours and abdominal organs and tissues between VNC and TNC images in patients undergoing DL-CT enhanced examinations and showed that low-keV VMIs from the nephrographic phase may provide almost as much enhancement and lesion visibility information about tumours as corticomedullary images when diagnosing RCC. The utilisation of VNC images and low-keV VMIs with DL-CT to replace TNC and corticomedullary phase images may be feasible and could lead to substantial reduction in the radiation dose for patients with RCC.

## Data Availability

The datasets used and analysed during the current study are available from the corresponding author on reasonable request.

## Abbreviations

CTDlvol: The volume CT dose index
DECT: Dual-energy CT
DL-CT: Dual-layer detector spectral CT
DLP: Dose–length product
dsDECT: Dual-source dual-energy systems,
ED: Effective dose
RCC: Renal cell carcinoma
ROI: Region of interest
rsDECT: Single-source rapid KV switching systems
SNR: Signal-to-noise ratio
TNC: True non-contrast
VMI: Virtual monoenergetic image
VNC: Virtual non-contrast

## References

[1] Dunnick NR (2016). Renal cell carcinoma: staging and surveillance. Abdom Radiol (NY).

[2] Ljungberg B, Albiges L, Abu-Ghanem Y (2019). European Association of Urology Guidelines on Renal Cell Carcinoma: the 2019 update. Eur Urol.

[3] Hsieh JJ, Purdue MP, Signoretti S (2017). Renal cell carcinoma. Nat Rev Dis Primers.

[4] Escudier B, Eisen T, Porta C (2012). Renal cell carcinoma: ESMO Clinical Practice Guidelines for diagnosis, treatment and follow-up. Ann Oncol.

[5] Warren AY, Harrison D (2018). WHO/ISUP classification, grading and pathological staging of renal cell carcinoma: standards and controversies. World J Urol.

[6] Gray RE, Harris GT (2019). Renal cell carcinoma: diagnosis and management. Am Fam Physician.

[7] Cheng K, Cassidy F, Aganovic L, Taddonio M, Vahdat N (2019). CT urography: how to optimize the technique. Abdom Radiol (NY).

[8] Higaki T, Nakamura Y, Fukumoto W, Honda Y, Tatsugami F, Awai K (2019). Clinical application of radiation dose reduction at abdominal CT. Eur J Radiol.

[9] Mohammadinejad P, Ehman EC, Vasconcelos RN (2020). Prior iterative reconstruction (PIR) to lower radiation dose and preserve radiologist performance for multiphase liver CT: a multi-reader pilot study. Abdom Radiol (NY).

[10] Yecies T, Bandari J, Macleod L, Fam M, Davies BJ, Jacobs BL (2019). Evaluation of the risks and benefits of computed tomography urography for assessment of gross hematuria. Urology.

[11] Mccollough CH, Leng S, Yu L, Fletcher JG (2015). Dual- and multi-energy CT: principles, technical approaches, and clinical applications. Radiology.

[12] Rassouli N, Etesami M, Dhanantwari A, Rajiah P (2017). Detector-based spectral CT with a novel dual-layer technology: principles and applications. Insights Imaging.

[13] Megibow AJ, Kambadakone A, Ananthakrishnan L (2018). Dual-energy computed tomography: image acquisition, processing, and workflow. Radiol Clin N Am.

[14] Grajo JR, Sahani DV (2018). Dual-energy CT of the abdomen and pelvis: radiation dose considerations. J Am Coll Radiol.

[15] Albrecht MH, Vogl TJ, Martin SS (2019). Review of clinical applications for virtual monoenergetic dual-energy CT. Radiology.

[16] Jamali S, Michoux N, Coche E, Dragean CA (2019). Virtual unenhanced phase with spectral dual-energy CT: is it an alternative to conventional true unenhanced phase for abdominal tissues?. Diagn Interv Imaging.

[17] Ananthakrishnan L, Rajiah P, Ahn R (2017). Spectral detector CT-derived virtual non-contrast images: comparison of attenuation values with unenhanced CT. Abdom Radiol (NY).

[18] Meyer M, Nelson RC, Vernuccio F (2019). Virtual unenhanced images at dual-energy CT: influence on renal lesion characterization. Radiology.

[19] Ma G, Han D, Dang S (2021). Replacing true unenhanced imaging in renal carcinoma with virtual unenhanced images in dual-energy spectral CT: a feasibility study. Clin Radiol.

[20] Fulton N, Rajiah P (2018). Abdominal applications of a novel detector-based spectral CT. Curr Probl Diagn Radiol.

[21] Sauter AP, Muenzel D, Dangelmaier J (2018). Dual-layer spectral computed tomography: virtual non-contrast in comparison to true non-contrast images. Eur J Radiol.

[22] Graser A, Johnson TR, Hecht EM (2009). Dual-energy CT in patients suspected of having renal masses: can virtual nonenhanced images replace true nonenhanced images?. Radiology.

[23] Reimer RP, Flatten D, Lichtenstein T (2019). Virtual monoenergetic images from spectral detector CT enable radiation dose reduction in unenhanced cranial CT. AJNR Am J Neuroradiol.

[24] Hickethier T, Kroeger JR, Lennartz S, Doerner J, Maintz D, Chang DH (2020). Venous-phase chest CT with reduced contrast medium dose: Utilization of spectral low keV monoenergetic images improves image quality. Eur J Radiol.

[25] Tsang DS, Merchant TE, Merchant SE, Smith H, Yagil Y, Hua CH (2017). Quantifying potential reduction in contrast dose with monoenergetic images synthesized from dual-layer detector spectral CT. Br J Radiol.

[26] Zopfs D, Laukamp KR, Pinto Dos Santos D (2019). Low-keV virtual monoenergetic imaging reconstructions of excretory phase spectral dual-energy CT in patients with urothelial carcinoma: a feasibility study. Eur J Radiol.

